# A Manual of Midwifery

**Published:** 1874-01

**Authors:** 


*A Manual of Midwifery.* By Dr. Karl Schrœder. Translated from the Third German Edition. By Dr. Charles H. Carter (London). New York: D. Appleton & Co., 1873. Buffalo: Martin Taylor.

Dr. Lieshman's object in preparing this work was, he says "to furnish Student's and Practitioners a Complete System of Midwifery of the present day." That he has done this thoroughly and in a manner which for completeness of style will form a favorable contrast with some of the works which have been placed before the profession; all who have had the pleasure of reading his work will at once admit. To qualify himself for this work our author tells us that he has made himself familiar with most of the modern works upon the subject, both British and foreign. Of this statement his book gives ample testimony in most instances of the truth. We are familiar with no treatise upon Obstetrics, which is so fully in accordance with, and evinces so thorough knowledge of the accepted teaching of the present time.

Although the work is in most respects an admirable one, we can not but regret that this American Edition did not pass through the hands of an American Editor, in order to make it conform with our usages and teachings. We have noticed a few passages which are to be accepted with a degree of allowance, and others which might have been somewhat improved. Our space does not allow us to notice these in detail as we should desire. We can only mention as an Example those passages relating to the forceps and their application which are among the most faulty in the book.

The student of midwifery looks upon the use of the forceps as one of the most formidable operations in obstetric practice and will naturally look to a work of this kind for the latest and most advanced knowledge. In this respect we are sorry to say that he will be disappointed, for Prof. Liesbman treats this important topic in a hesitating and uncertain tone.

The subject of inversion of the uterus and its treatment receives somewhat extented notice at the hands of the author. In cases of chronic inversion he recommends the use of taxis, replacing that part first which came down last, giving to Drs. Montgomery and McClintock the credit of the suggestion. He speaks of a case of Tyler Smith's in which he reduced an inverted uterus of *ten* years duration, evidently refering to the case reported in the *Medical Times and Gazette* for April 24, 1858, and which had been *twelve* years inverted. In this connection he entirely ignores the labors of Prof. White, whose claims for priority in the employment and scientific description of taxis are well founded, and seems entirely ignorant of his having reduced in the eleven cases which he has successfully treated, two which were of the unprecedented time of *fifteen* and *twenty-two* years duration.

The few passages such as have been mentioned are however more than counterbalanced by the general excellence of the work, and we predict that it will be, as it deserves to, a general favorite with the profession. The typography is excellent and the illustrations well chosen and in many instances entirely new.

To meet a want which the work of Naegele, hitherto the standard German authority, did not supply, Dr. Schrœder published in 1870 the first edition of this Manual of Obstetrics. Two succeeding editions were called for and it is the last of these that the English translation is made. The work is intended to be a practical one and hence omits all discussion of points not yet fully established. The condensed style of the author, who takes it for granted that the elementary points are already understood, will render it unfitting as a text-book. As a manual of the subject to be referred to by the advanced practitioner it will be fully appreciated. The anatomy of the pelvis and female organs of generation is omitted, the reader being referred to anatomical works for its study. As an exponent of German teaching it will find a ready reception and is worthy of the careful attention of every medical practitioner.

